# Trm7/FTSJ1-Mediated tRNA Anticodon-Loop 2′-O-Methylation: From Structural Mechanisms to Translational Dysfunction and Disease

**DOI:** 10.3390/genes17060697

**Published:** 2026-06-15

**Authors:** Huan Sheng, Jun Yao

**Affiliations:** 1 State Key Laboratory (SKL) of Biobased Transportation Fuel Technology, Ocean College, Zhejiang University, Zhoushan 321002, China; 2 Hainan Institute, Zhejiang University, Sanya 572025, China

**Keywords:** Trm7/FTSJ1, tRNA modification, 2′-O-methylation, anticodon stem-loop, tRNA^Phe^, codon-biased translation, neurodevelopmental disease

## Abstract

Transfer RNAs (tRNAs) are chemically matured decoding molecules that are central to protein synthesis. Their post-transcriptional modifications, especially those in the anticodon stem-loop (ASL), shape local RNA structure, codon recognition and translational fidelity at the tRNA-mRNA decoding interface. 2′-O-methylation (Nm) is a conserved ribose modification installed at selected ASL positions, particularly positions 32 and 34, by the modular Trm7/FTSJ1 methyltransferase system. Rather than directly changing base-pairing identity, these marks help prepare the decoder for efficient translation and function within an interconnected 32–34–37 modification network, best illustrated by tRNA^Phe^. Loss of Trm7/FTSJ1-mediated Nm may impair selected codon–tRNA decoding pairs; in yeast, Trm7 deficiency is additionally associated with GAAC activation and phenotypes consistent with reduced functional tRNA^Phe^ availability. In humans, mutations in FTSJ1 are associated with nonsyndromic X-linked intellectual disability (NSXLID), suggesting that disruption of tRNA chemical maturation can affect neuronal translation programs. In this review, we integrate anticodon-loop modifications at positions 32, 34, and 37 into a decoder-centered framework and compare the conserved enzymatic logic of yeast Trm7 and human FTSJ1 with their divergent substrate repertoires. By synthesizing structural, biochemical, genetic, and translational evidence, we distinguish established mechanisms from working models and unresolved questions concerning tRNA modification hierarchies and neuronal vulnerability.

## 1. Introduction

Transfer RNAs (tRNAs) are chemically matured decoding molecules, not merely passive adaptors in protein synthesis [1,2]. Among tRNA modifications, those in the anticodon stem-loop (ASL) are particularly important because the ASL operates at the immediate interface between tRNA, mRNA codons and the ribosomal decoding center [3]. Anticodon-loop modifications regulate translation by shaping ASL conformation, codon recognition and reading-frame maintenance [4,5,6]. 2′-O-methylation (Nm) is a conserved ribose modification that shapes local RNA structure and flexibility [7]. In the tRNA anticodon loop, Nm acts on the decoder itself without directly altering base-pairing identity. How this local chemical tuning is converted into altered decoding, cellular dysfunction and disease remains a central question.

The yeast methyltransferase Trm7 and its human homolog FTSJ1 provide a useful model for addressing this question. Trm7/FTSJ1-dependent Nm occurs at specific target sites, particularly positions 32 and/or 34 of selected tRNAs, linking anticodon-loop chemical maturation to substrate-specific decoding [8,9]. Mutations in the human *FTSJ1* gene have been linked to nonsyndromic X-linked intellectual disability (NSXLID), placing this conserved modification pathway in a disease-relevant context [9,10]. Natural modifications in the anticodon domain can preorganize tRNAs for cognate and wobble codon binding [11]. This concept suggests that chemical preorganization of the tRNA anticodon loop is not merely a housekeeping feature, but may contribute to translation homeostasis in disease-relevant settings. Although individual aspects of FTSJ1 function and tRNA 2′-O-methylation have been addressed in previous studies, a synthesis connecting positional modification logic, enzyme-cofactor specificity, substrate vulnerability, translational consequences, and disease relevance remains needed. In this review, we integrate Trm7/FTSJ1-dependent Nm at positions 32 and 34 with neighboring anticodon-loop modifications, including position 37, within a decoder-centered framework. By comparing the conserved enzymatic logic and divergent substrate repertoires of yeast Trm7 and human FTSJ1, and by synthesizing structural, biochemical, genetic, and translational evidence, we distinguish established mechanisms from working models and unresolved questions concerning tRNA modification hierarchies and neuronal vulnerability.

## 2. Positional Logic of Anticodon-Loop Nm

The regulatory capacity of anticodon-loop modifications is strongly shaped by their spatial position within the anticodon stem-loop (ASL) [12]. The anticodon itself comprises nucleotides 34–36, with nucleotide 34 serving as the wobble position. Around the anticodon triplet, positions 32, 34 and 37 form a functionally integrated module that helps prepare the ASL for entry into the ribosomal aminoacyl (A) site: position 32 contributes to local architecture and flexibility, position 34 governs wobble codon recognition [13,14], and position 37 stabilizes the codon–anticodon mini-helix [11] and supports reading-frame maintenance [4,6,15]. Within this spatial framework, 2′-O-methylation (Nm) has a distinct biophysical role because it modifies the ribose 2′-OH rather than the base-pairing atoms of the nucleotide. Cm32 and Nm34 can modulate decoding efficiency and fidelity by shaping sugar pucker, local rigidity, base stacking and backbone geometry, while leaving Watson–Crick base pairing and codon identity unchanged [5,7,16].

Importantly, the 32–34–37 module functions as an interconnected modification network rather than as a set of isolated chemical marks. Eukaryotic tRNA^Phe^ provides the clearest example: Trm7/FTSJ1-dependent Cm32 and Gm34 are functionally linked to the formation of wybutosine-related modifications at position 37 in yeast and human systems [8,9,17]. This coupling is consistent with a model in which loss of anticodon-loop Nm may affect more than a single methyl mark by destabilizing a broader modification network and compromising the structural readiness of the decoder. This positional logic also helps define the specific role of the Trm7/FTSJ1 pathway within the broader landscape of Nm-mediated translational regulation. Across the translation apparatus, tRNA Nm acts on decoders, rRNA Nm acts on the ribosomal machinery, and mRNA Nm acts on translation templates (Table 1).

## 3. Conserved and Structural Mechanisms of the Trm7/FTSJ1 System

Site specificity in the Trm7/FTSJ1 system is achieved through a modular mechanism. The catalytic 2′-O-ribose methyltransferase does not act alone; instead, partner proteins guide it to particular positions within selected tRNA anticodon loops. This allows the same catalytic core to modify different nucleotides, especially positions 32 and 34. Thus, Trm7/FTSJ1-mediated Nm formation is best understood as a cofactor-guided tRNA recognition and positioning system [25], rather than as a standalone methyltransferase reaction [8,18,25]. This modular logic is most clearly illustrated by the yeast Trm7 system.

### 3.1. Yeast Trm7-Trm732/Trm734 Module

The yeast system provides a clear genetic and biochemical model for how partner proteins confer positional specificity on Trm7 [26,27]. In *Saccharomyces cerevisiae*, Trm7 relies on two distinct partners: Trm732 supports efficient 2′-O-methylation at position 32 [28], such as Cm32, whereas Trm734 promotes Nm formation at position 34 [29], including wobble-position modifications such as Gm34 [8]. This division of labor is supported by complementary evidence. Affinity purification confirmed that Trm7 physically associates with Trm732 and Trm734; nucleoside analysis of purified tRNAs showed that loss of Trm732 preferentially affects C32 methylation, whereas loss of Trm734 primarily impairs 2′-O-methylation at position 34; and genetic analysis showed that *trm732*Δ *trm734*Δ double mutants resemble *trm7*Δ mutants more strongly than either single mutant [8]. Together, these data indicate that Trm732 and Trm734 are not merely binding partners, but specificity factors that help determine which anticodon-loop nucleotide is presented to the Trm7 catalytic center. This yeast module establishes a principle that is also used, with adaptations, in the human FTSJ1 system.

### 3.2. Human FTSJ1-THADA/WDR6 Module

The human FTSJ1 system preserves the same general modular logic, while showing important differences in substrate selection [30]. FTSJ1 acts as the core catalytic enzyme for anticodon-loop Nm formation, but its position-specific activity depends on accessory proteins. Notably, CRISPR-Cas9 knockout of FTSJ1 in human cells results in the simultaneous loss of methylation at both positions 32 and 34, including Cm32 and Gm34 in tRNA^Phe^, as shown by mass spectrometry [18]. By contrast, *WDR6* knockout selectively abolishes Nm34 while leaving position-32 methylation largely intact, indicating that WDR6 functions as the site-directing factor for wobble-position methylation [18]. In parallel, THADA cooperates with FTSJ1 to promote Nm32 formation across multiple tRNA species [9,17,25]. These findings show that the human pathway retains the “core enzyme plus site-directing cofactor” strategy, although its substrate spectrum is not identical to that of yeast. They also raise the key structural question for the next section: how do these cofactors position the correct nucleotide for methyl transfer?

### 3.3. Structural Basis of Site-Specific tRNA Recognition

The structural basis of site specificity in the Trm7/FTSJ1 system begins with a simple problem: the catalytic core alone is unlikely to distinguish neighboring nucleotides within a flexible anticodon loop. Like other SAM-dependent 2′-O-ribose methyltransferases [31], FTSJ1 contains a Rossmann-like fold that binds S-adenosylmethionine (SAM) as the methyl donor and catalyzes methyl transfer to the target ribose 2′-OH [32,33,34]. However, this catalytic fold must be coupled to a mechanism that selects and positions the correct nucleotide near the active site [25,35].

Accessory proteins provide this positioning mechanism. Structural and biochemical evidence indicates that THADA expands the tRNA-binding surface of FTSJ1 and helps orient the substrate so that position 32 can access the catalytic center [25,36]. For Nm34 formation, equivalent high-resolution structural information is still limited, but biochemical reconstitution supports a site-directing role for WDR6 in wobble-position methylation [18]. Thus, specificity in the Trm7/FTSJ1 system is not encoded solely in the methyltransferase domain; it emerges from the coordinated action of the catalytic core, accessory protein-guided RNA recognition and spatial presentation of the target ribose [37,38]. This structural logic explains how one catalytic enzyme can be directed to different anticodon-loop positions, while also raising the question of how substrate selection diverged across species.

### 3.4. Conservation and Divergence from Yeast to Humans

**Figure 1:**
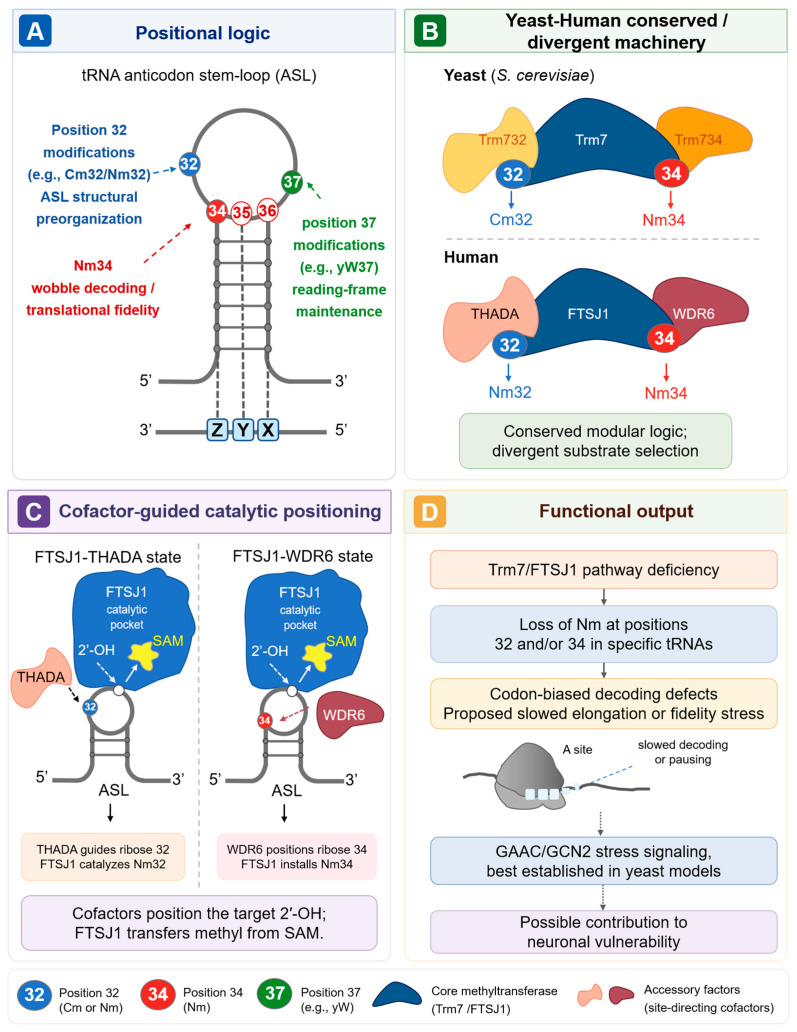
Decoder-centered model of Trm7/FTSJ1-mediated anticodon-loop 2′-O-methylation. (**A**) Positions 32, 34, and 37 form a chemically tunable module within the tRNA anticodon stem-loop. Cm/Nm32 contributes to ASL structural preorganization, Nm34 modulates wobble decoding and translational fidelity, and position-37 modification, such as yW37 in tRNA^Phe^, support reading-frame maintenance; (**B**) the Trm7/FTSJ1 pathway follows a conserved modular logic with species-specific cofactors and substrates. In yeast, Trm7 cooperates with Trm732 for Cm32 formation and with Trm734 for Nm34 formation. In humans, FTSJ1 cooperates with THADA for Nm32 formation and with WDR6 for Nm34 formation; (**C**) site-specific catalysis depends on cofactor-guided positioning of the target ribose 2′-OH near the Trm7/FTSJ1 catalytic pocket. SAM provides the methyl donor, whereas THADA/Trm732-like or WDR6/Trm734-like partners direct modification at position 32 or 34, respectively; (**D**) loss of anticodon-loop 2′-O-methylation at positions 32 and/or 34 may disrupt specific tRNA modification circuits. Codon-biased decoding defects have been observed in human cells, whereas GCN2-dependent stress activation is best established in yeast Trm7-deficient models [18,40,41]. Dashed arrows indicate proposed or incompletely established links, including slowed elongation, fidelity stress, and the possible contribution of these pathways to neuronal vulnerability. Numbers 32, 34, and 37 indicate tRNA nucleotide positions; Z, Y, and X indicate codon bases paired with anticodon positions 34, 35, and 36. Blue, red, and green denote positions 32, 34, and 37, respectively; enzyme and cofactor shapes are defined in the bottom legend.

The Trm7/FTSJ1 pathway illustrates a balance between conserved architecture and divergent substrate selection. In both the yeast Trm7-Trm732/Trm734 system and the human FTSJ1-THADA/WDR6 system, a core methyltransferase relies on distinct partner proteins to achieve position-specific anticodon-loop Nm formation [8,9,17,18]. However, conserved machinery does not mean identical targets. Yeast Trm7 modifies tRNA^Phe^, tRNA^Trp^ and tRNA^Leu(UAA)^, whereas human FTSJ1-dependent Nm34 formation involves a partly distinct set of substrate tRNAs. Similarly, WDR6 is better described as a functional counterpart of Trm734 than as a strict ortholog. This conservation-with-divergence framework is important for interpreting human phenotypes; yeast provides the core biochemical logic, but human disease contexts must be understood in light of mammalian substrate selection, tissue-specific tRNA expression and codon-usage programs [39]. Yeast models therefore provide a powerful framework for defining the biochemical and genetic logic of the Trm7 system, but they should not be regarded as direct models of mammalian neuronal physiology. Differences in substrate repertoires, cell-type complexity, transcriptome composition, and translational demands mean that disease-related mechanisms inferred from yeast require direct validation in mammalian neuronal systems. This point naturally leads to the next question: which substrate tRNAs are most vulnerable to Trm7/FTSJ1 deficiency? This conserved modular logic and its translational consequences are summarized in Figure 1.

## 4. Substrate Vulnerability: tRNA^Phe^ Dominance and Divergent Substrate Selection

The effects of Trm7/FTSJ1-mediated anticodon-loop Nm are not uniform across the tRNA pool. Although this pathway modifies a selected subset of tRNAs, different substrates contribute unequally to cellular phenotypes, so the effects are substrate-biased rather than uniform. This hierarchy is most evident in yeast, where tRNA^Phe^ dominates the Trm7-associated growth phenotype, but becomes more complex in mammals, where FTSJ1 substrate selection has diverged. These observations support a substrate-biased framework in which the consequences of Trm7/FTSJ1 deficiency depend on the affected tRNA repertoire and experimental system [8,18,41].

### 4.1. tRNA^Phe^ Dominance and the Yeast Substrate Hierarchy

Among yeast Trm7 substrates, tRNA^Phe(GAA)^ is the best-supported functionally vulnerable target [8,41]. Loss of Trm7-dependent Cm32 and Gm34 impairs yW37 formation in this tRNA, linking these modifications within an interconnected anticodon-loop circuit [8,17,42]. Related defects in position-37 modification have also been reported in human FTSJ1-deficient cells [9,18]. At the same time, yeast Trm7 is not a single-target enzyme. Nucleoside analysis shows that Trm7 is also required for 2′-O-methylation at positions 32 and 34 of tRNA^Trp(CCA)^ and tRNA^Leu(UAA)^.

However, a clear functional hierarchy exists among these substrates. In yeast *trm7*Δ mutants, steady-state tRNA^Phe^ levels are largely maintained, yet cells display severe growth defects and GAAC activation. These phenotypes are suppressed by overexpression of mature tRNA^Phe^, phenylalanyl-tRNA synthetase, or eEF1A, whereas available rescue data do not support comparable rescue by other yeast Trm7 substrates [41]. Thus, in yeast, Trm7 has a broader biochemical substrate range, whereas the observed growth and stress-response phenotypes appear to be particularly sensitive to the functional competence of the tRNA^Phe^ pool. This yeast hierarchy raises the next question: does the mammalian system preserve the same dependency, or has substrate selection been remapped during evolution?

### 4.2. Mammalian Substrate Divergence and Unresolved Substrate Hierarchy

Human FTSJ1 substrate specificity cannot be directly extrapolated from the yeast Trm7 system, indicating evolutionary divergence in target selection [43]. In yeast, Trm7 modifies not only tRNA^Phe^ but also tRNA^Trp(CCA)^ and tRNA^Leu(UAA)^. In human cells, however, FTSJ1-dependent Nm34 formation has been linked to tRNA^Phe(GAA)^ and tRNA^Leu(CAA)^, whereas the classical yeast substrates tRNA^Trp(CCA)^ and tRNA^Leu(UAA)^ do not appear to be equivalent FTSJ1-WDR6 targets in the same experimental contexts [18]. This shift indicates that the core modular architecture of the pathway is conserved, while the substrate landscape has diverged across species. Whether tRNA^Phe^ remains the dominant vulnerable target in mammals, or whether neuronal cells rely on a distinct tissue-specific hierarchy of FTSJ1-sensitive tRNAs, remains unresolved [43,44]. This question is particularly relevant because mammalian tissues show substantial differences in mature tRNA expression and modification patterns [45]. Thus, the yeast substrate hierarchy provides a useful mechanistic framework but may not predict the dominant tRNA targets or translational consequences in mammalian neurons.

## 5. Translational Consequences: From Codon-Biased Decoding to GAAC/GCN2 Signaling

Trm7/FTSJ1-mediated anticodon-loop Nm is not a passive chemical mark on mature tRNAs; it contributes to the functional maturation of selected decoding tRNAs. Available evidence supports selective rather than uniform translational consequences of Trm7/FTSJ1 deficiency. Codon-biased decoding defects have been observed in human cells and Ftsj1-deficient mouse brain, whereas reduced functional tRNA^Phe^ availability and GAAC activation are best established in yeast models [18,41,44]. These effects may converge on altered elongation dynamics and stress signaling, although their relative contributions may depend on the affected tRNA, codon context, species, and cellular state.

### 5.1. Codon-Biased Decoding Defects

A key measurable consequence of Trm7/FTSJ1 deficiency is codon-biased decoding rather than generalized translation collapse. In human cells, ribosome profiling and targeted reporter assays show that loss of FTSJ1 reduces translation efficiency at the UUU codon decoded by tRNA^Phe(GAA)^, whereas the synonymous UUC codon is less affected in the same experimental context [18]. In vivo, ribosome profiling of Ftsj1-deficient mouse brain similarly reveals codon-specific changes in translation efficiency, accompanied by neurological and behavioral abnormalities [44,46]. These findings indicate that Trm7/FTSJ1-dependent Nm marks help optimize specific codon–tRNA decoding pairs, making mRNAs enriched in vulnerable codons more susceptible to translational imbalance [43,47]. The next question is how loss of a ribose methyl group in the ASL alters decoding dynamics at the ribosomal A site.

### 5.2. A-Site Decoding Dynamics and Fidelity Stress

How anticodon-loop Nm loss specifically alters decoding at the ribosomal A site remains incompletely defined. Because Nm modifies the ribose 2′-OH rather than the Watson–Crick base-pairing edge, loss of Cm32 or Nm34 is unlikely to directly alter base-pairing identity [7,48]. Instead, it is more likely to affect ASL preorganization, local rigidity or accommodation dynamics, thereby lowering the decoding efficiency of selected hypomodified tRNAs [49,50]. Although the precise kinetic step impaired by Trm7/FTSJ1 deficiency remains to be resolved, altered decoding dynamics could increase the risk of local ribosome pausing or fidelity stress at susceptible codons [51,52]. This possibility is consistent with broader evidence that anticodon-loop modifications [53,54], particularly around positions 34 and 37, contribute to codon–anticodon stability, reading-frame maintenance [55,56,57] and efficient elongation [6]. However, direct evidence for widespread ribosome pausing [58,59], frameshifting [60,61], or proteome-wide mistranslation, particularly in mammalian FTSJ1-deficient neurons, remains limited. Therefore, these effects should be regarded as possible, context-dependent downstream consequences rather than established general outcomes.

### 5.3. Functional tRNA Availability and GAAC/GCN2 Activation

Evidence that Trm7 deficiency reduces the functional competence of mature tRNAs is strongest in yeast. In yeast *trm7*Δ mutants, steady-state tRNA^Phe^ levels are largely maintained, yet cells exhibit growth defects, eIF2α phosphorylation, and GAAC activation. These phenotypes are strongly suppressed by overexpression of mature tRNA^Phe^, phenylalanyl-tRNA synthetase, or eEF1A [41]. Rather than indicating a quantitative loss of tRNA^Phe^, these findings support a model in which the properly modified tRNA^Phe^ pool has reduced functional competence, consistent with broader evidence that tRNA modification defects can perturb translational stress responses [62,63,64,65,66]. GCN2 can sense tRNA-related translational stress through mechanisms involving its HisRS-like domain and activation of the kinase domain [62,63,64,67]. Subsequent eIF2α phosphorylation links this stress signal to broader translational regulation and cellular adaptation [68,69]. However, whether comparable loss of functional tRNA availability and GCN2/eIF2α activation occurs in mammalian FTSJ1-deficient tissues remains to be established.

Interpreting this pathway requires separating direct observations from broader mechanistic inferences [70,71]. Table 2 summarizes the main links between anticodon-loop Nm loss, codon-biased decoding, functional tRNA availability and downstream stress responses [72,73], while highlighting unresolved questions for each step. This evidence map provides a cautious basis for connecting Trm7/FTSJ1-dependent tRNA hypomodification to neuronal dysfunction and disease.

## 6. Disease Relevance: FTSJ1, Neuronal Vulnerability and Tissue-Specific Translation

Among the biological consequences of Trm7/FTSJ1 deficiency, the clearest organismal phenotype is neurodevelopmental disease [76]. The central question is not simply whether FTSJ1 is disease-associated, but why defects in this conserved tRNA modification enzyme preferentially affect cognition and neuronal function.

### 6.1. Human FTSJ1 Mutations and NSXLID

Human genetics first established the disease relevance of FTSJ1. Loss-of-function and splice-site mutations in human FTSJ1 were identified in families with NSXLID [77,78,79], implicating this methyltransferase in cognitive development [10,80]. Subsequent biochemical studies linked these variants to impaired 2′-O-methylation at positions 32 and/or 34 of selected tRNAs, particularly tRNA^Phe^, often accompanied by defects in position-37 modification [9,18]. Although NSXLID remains the best-established human disease association, FTSJ1 has also been implicated in non-neurodevelopmental contexts such as NSCLC [81]. These findings suggest that FTSJ1-associated disease is better viewed as a selective disruption of tRNA modification circuits, rather than as a uniform translation disorder [82]. Such disruption may preferentially affect vulnerable codon–tRNA decoding programs.

### 6.2. Brain-Specific Codon-Biased Translation Defects

Animal models strengthen the link between FTSJ1-dependent tRNA Nm and brain-relevant translational regulation. In Ftsj1-deficient mice [83,84], loss of the enzyme perturbs codon-specific translation efficiency in the brain and is associated with memory-related and anxiety-like behavioral abnormalities [44]. Additional patient-derived and model-system studies connect FTSJ1 loss to altered tRNA Nm profiles, deregulated disease-associated genes [85], abnormal neurite or spine-like morphology and learning defects [43]. Together, these findings support the possibility that FTSJ1 deficiency selectively alters neuronal translation programs. However, the extent to which the underlying mechanisms parallel those defined in yeast remains uncertain.

### 6.3. Why Neurons Are Vulnerable

Neuronal vulnerability to FTSJ1 deficiency may reflect the convergence of neural cell biology and tissue-specific translation demands [86]. Neurons rely heavily on spatially and temporally controlled local translation in dendrites [87,88], axons and synapses [89], where protein synthesis must support activity-dependent plasticity and continuous synaptic remodeling [90,91,92]. As long-lived, highly polarized cells, neurons face sustained proteostatic pressure and may be particularly vulnerable to chronic low-level translation stress because they cannot easily dilute damaged or misfolded proteins through cell division [93]. A further layer of vulnerability may come from brain-specific codon–tRNA usage. If neuronal transcripts are enriched for codons decoded by FTSJ1-sensitive tRNAs, they may be especially affected by FTSJ1 deficiency. However, systematic enrichment of FTSJ1-sensitive codons in neuronal or synaptic transcript sets has not yet been demonstrated and remains hypothetical. This hypothesis nevertheless provides a testable framework for linking a broadly expressed tRNA modification enzyme to tissue-selective neurodevelopmental phenotypes [86,94]. Mechanisms defined in yeast should not be assumed to operate identically in mammalian neurons, which differ in tRNA expression profiles, transcriptome composition, cellular architecture, and translational demands. Future studies in mammalian neuronal models should determine whether FTSJ1-sensitive codons are enriched in transcripts involved in synapse formation [95], dendritic spine remodeling [96], neuronal proteostasis [97,98] or activity-dependent plasticity [99].

### 6.4. FTSJ1 Beyond NSXLID

Beyond NSXLID, emerging evidence implicates FTSJ1 in broader regulatory and pathological contexts. In *Drosophila*, FTSJ1-related tRNA 2′-O-methylation has been linked to small-RNA silencing [39]. In non-small-cell lung cancer models, FTSJ1 has been associated with tRNA 2′-O-methyladenosine modification, DRAM1 regulation, and malignant phenotypes [81]. These findings suggest that FTSJ1-dependent tRNA modification may influence gene-expression programs in a context-dependent manner. However, the relevant tRNA substrates, downstream mechanisms, and clinical significance outside neurodevelopmental disease remain incompletely defined. Thus, although NSXLID remains the best-established human disease association of FTSJ1, its biological significance may extend to broader physiological and pathological settings.

### 6.5. Limitations and Alternative Interpretations

Codon-biased decoding provides one possible explanation for the consequences of FTSJ1 deficiency, but it is unlikely to represent the only relevant mechanism. Altered tRNA stability or maturation, including rapid tRNA decay (RTD)-like processes, may contribute to some phenotypes, although direct evidence for such mechanisms in Trm7/FTSJ1 substrates remains limited [49,72,75]. FTSJ1 deficiency may also alter cellular stress adaptation and broader gene-expression programs rather than acting exclusively through specific codon–tRNA pairs [41,70,85]. In neurons, tissue-specific tRNA expression, local translation, long cellular lifespan, and sustained proteostatic demands may further influence vulnerability [86,87,88,89,90,91,92,93,97,98]. These mechanisms are not mutually exclusive.

Differences among experimental systems also argue against a single uniform disease pathway. In yeast, tRNA^Phe^-associated growth and GAAC phenotypes can be suppressed by increasing tRNA^Phe^ pathway capacity [8,41], whereas human cells exhibit a partly divergent FTSJ1 substrate repertoire [18,43], and Ftsj1-deficient mouse brain shows codon-specific translational changes [44]. Thus, the yeast substrate hierarchy and stress-response phenotypes provide mechanistic guidance but should not be regarded as direct explanations of human neuronal dysfunction. Moreover, direct evidence for widespread ribosome pausing, frameshifting, or translational fidelity defects in mammalian FTSJ1-deficient neurons remains limited, and systematic enrichment of FTSJ1-sensitive codons in neuronal or synaptic transcripts has not yet been established. Accordingly, the decoder-centered framework presented here should be regarded as a working model rather than a fully established causal pathway.

## 7. Conclusions and Future Perspectives

Research on the Trm7/FTSJ1 methyltransferase system has progressed from yeast genetics to mechanistic studies of tRNA modification, codon-biased decoding, and human neurodevelopmental disease [100,101,102]. A central concept emerging from these studies is that anticodon-loop 2′-O-methylation is not simply a passive chemical decoration on mature tRNAs, but part of a broader modification network that can influence decoding capacity, functional tRNA availability, and cellular translation programs. By considering Trm7/FTSJ1-dependent Nm at positions 32 and 34 together with neighboring anticodon-loop modifications, including position 37, this review highlights a decoder-centered framework in which conserved enzymatic principles are combined with species-specific substrate repertoires. At the same time, the available evidence also indicates that several proposed links between FTSJ1 deficiency, translation-related defects, and neuronal dysfunction remain incompletely established and require further experimental validation.

Several unresolved questions define the next stage of the field. First, the mammalian substrate hierarchy remains unclear: does tRNA^Phe^ remain the dominant vulnerable substrate across tissues, or do distinct cell types rely on different FTSJ1-sensitive tRNAs [103]? Second, the full range of codons and transcripts affected by Nm loss needs to be mapped by integrating tRNA modification profiling, ribosome profiling, codon-enrichment analysis and quantitative proteomics. Third, the basis of neuronal vulnerability should be tested in specific brain regions, cell types and developmental stages, with attention to local translation, synaptic protein turnover [104,105] and proteostatic stress.

Addressing these questions will require moving beyond single-readout assays toward integrated models that connect RNA chemistry, tRNA substrate selection, codon-specific translation and cellular physiology. The Trm7/FTSJ1 pathway provides a useful model for understanding how small chemical changes in the tRNA anticodon loop may shape translation programs and potentially contribute to tissue-selective disease. More broadly, it illustrates how RNA chemical maturation can act as a regulatory layer between the genetic code and cellular phenotype.

## Figures and Tables

**Table 1:** Nm regulation across the translation apparatus.

RNA Species	Conceptual Role in Translation	Major Modification System(s)	Main Regulatory Effects	Relevance
tRNA Nm	Decoders [6,8,9,18]	Trm7/FTSJ1 and other tRNA 2′-O-methyltransferases	Modulates ASL structure, tRNA functional availability, codon recognition, decoding efficiency, fidelity and reading-frame maintenance	Direct decoder target
rRNA Nm	Machinery [19,20]	C/D box snoRNP-fibrillarin complexes	Supports ribosome biogenesis, ribosomal architecture, conformational dynamics, translational fidelity and ribosome heterogeneity	Modulates ribosomal machinery
mRNA Nm	Templates [21,22,23,24]	CMTR1/CMTR2-mediated cap-proximal Nm; selected snoRNA-guided internal Nm	Regulates mRNA cap function, processing, stability, innate immune recognition and transcript-specific translation	Acts on translation templates

**Table 2:** Evidence map linking Trm7/FTSJ1 deficiency to translational defects.

Mechanistic Link	Main Supporting System/Evidence	Unresolved Issue
tRNA^Phe^ hypomodification	Yeast *trm7*Δ cells and human FTSJ1-deficient cells [8,18]; loss/reduction in Cm32, Gm34 and position-37 wybutosine-related modifications [9,17]	Tissue-specific substrate hierarchy
Codon-biased decoding defect, especially UUU	Human cells and Ftsj1-deficient mouse brain; altered codon-specific translation efficiency [18,44]	Context dependence and full codon spectrum
Reduced functional tRNA^Phe^ availability	Yeast *trm7*Δ mutants; maintained tRNA^Phe^ abundance, GAAC activation and rescue by tRNA^Phe^, phenylalanyl-tRNA synthetase or eEF1A [41]	Conservation in mammalian tissues
GAAC/GCN2 activation	*S. cerevisiae* and *S. pombe*; eIF2α phosphorylation and GAAC activation [41,62,67]	Mammalian conservation in FTSJ1-deficient contexts
Ribosome pausing/slowed elongation	Codon-biased decoding defects are consistent with slowed elongation [5,55]	Direct codon-level pausing in Trm7/FTSJ1-deficient systems
Frameshifting/fidelity stress	ASL modification literature; 32–34–37 modification network and reading-frame maintenance [5,55,74]	Direct Trm7/FTSJ1-dependent causality
Altered tRNA stability or maturation, including rapid tRNA decay (RTD)	Evidence from other hypomodified tRNA systems [49,72,75]	Direct involvement of Trm7/FTSJ1 substrates remains unproven

## Data Availability

No new data were generated or analyzed in this study.

## Abbreviations

The following abbreviations are used in this manuscript:

ASL	anticodon stem-loop
Nm	2′-O-methylation
Cm32	2′-O-methylcytidine at position 32
Gm34	2′-O-methylguanosine at position 34
SAM	S-adenosylmethionine
GAAC	general amino acid control
GCN2	general control nonderepressible 2
eIF2α	eukaryotic initiation factor 2 alpha
NSXLID	nonsyndromic X-linked intellectual disability
RTD	rapid tRNA decay
snoRNP	small nucleolar ribonucleoprotein
CMTR1/CMTR2	cap methyltransferase 1/2
PheRS	phenylalanyl-tRNA synthetase
eEF1A	eukaryotic elongation factor 1A
yW37	wybutosine at position 37
o2yW37	peroxywybutosine at position 37
WDR6	WD repeat domain 6
THADA	thyroid adenoma-associated protein
